# Prevalence of paratuberculosis in cattle based on gross and microscopic lesions in Ethiopia

**DOI:** 10.1186/s12917-023-03725-x

**Published:** 2023-10-13

**Authors:** Temesgen Mohammed, Gezahegne Mamo, Aboma Zewude, Asegedech Sirak, Balako Gumi, Gobena Ameni

**Affiliations:** 1 Aklilu Lemma Institute of Pathobiology, Addis Ababa University, Addis Ababa, Ethiopia; 2 Department of Veterinary Medicine, College of Agriculture and Veterinary Medicine, United Arab Emirates University, Al Ain, United Arab Emirates; 3 College of Veterinary Medicine and Agriculture, Addis Ababa University, Bishoftu, Ethiopia; 4 National Animal Health Diagnostic and Investigation Center, Sebeta, Ethiopia

**Keywords:** Paratuberculosis, Prevalence, Pathology, Cattle, Ethiopia

## Abstract

**Background:**

Paratuberculosis, caused by *Mycobacterium avium* subsp. *paratuberculosis* (MAP), is a chronic progressive granulomatous enteritis mainly affecting domestic and wild ruminants worldwide. Although paratuberculosis could be prevail in Ethiopia, there is a scarcity of epidemiological data on paratuberculosis in the country. Thus, this study was conducted to estimate the prevalence of paratuberculosis based on gross and microscopic lesions in cattle slaughtered at ELFORA Abattoir, central Ethiopia. Small intestines and associated lymph nodes of 400 apparently healthy cattle which were slaughtered at ELFORA export abattoir were examined for gross and microscopic lesions of paratuberculosis. The microscopic lesions were classified into four grades (I-IV) based on the type and number of cells infiltrated into the lesion. The prevalence of paratuberculosis was estimated on the basis of gross as well as microscopic lesion of paratuberculosis.

**Results:**

The prevalence of paratuberculosis was 11.25% (95% Confidence interval, CI = 0.083–0.148) on the basis of gross lesion. However, relatively lower prevalence (2.0%, 95% CI = 0.01, 0.039) was recorded based on microscopic lesion. The gross lesions were characterized by intestinal thickening, mucosal corrugations and enlargement of associated mesenteric lymph nodes. On the other hand, the microscopic lesions were characterized by granuloma of different grades ranging from grade I to grade III lesions.

**Conclusions:**

The present study indicated the occurrence of paratuberculosis in cattle of Ethiopia based on the detection of gross and microscopic lesions consistent with the lesion of paratuberculosis. The result of this study could be used as baseline information for future studies on the epidemiology and economic significance of paratuberculosis.

## Background

Paratuberculosis (PTB), commonly known as Johne’s disease (JD), is a chronic progressive granulomatous enteritis of ruminants. It also affects a wide variety of domestic and wild life species worldwide [1, 2]. It is caused by *Mycobacterium avium* subsp. *paratuberculosis* (MAP) which is a facultative intercellular acid-fast bacillus (AFB) belonging to the genus *Mycobacterium*. MAP is an extremely slow growing mycobactin-dependent organism that replicates within the macrophages of both the gastrointestinal tract and associated lymphoid tissues [1]. Contaminated feed and water, bedding and soiled udders are thought to be the major routes for spread of the organism and young animals less than six months of age are thought to be the most susceptible to infection [3]. Cattle become infected early as young calves via faecal-oral transmission of the infectious agent [4]. Intrauterine infection in cattle has also been well documented [5].

Paratuberculosis induces a significant economic and health problem worldwide, especially in the cattle industry. Economic losses occur due to decreased in milk production (15–16%), low carcass yield [6, 7], decrease weight gain, increase infertility [3, 8], delay parturition [9] and premature culling of animals [10]. The economic loss attributed to the disease is estimated to be about $200 per infected cow per year in herd with at least 10% prevalence [11]. For example, a study conducted in the UK indicated that the average cost of paratuberculosis per year is $47 per a dairy cow and $31 per year per beef cattle [12]. Similarly, a study conducted in the United States reported an average economic loss of $22 to $27 per cow per year and total annual loss of $1.5 billion [13, 14].The disease is not only important because of its economic impact in the livestock industry but also it could be one of the causative agents of Crohn’s disease which is an inflammatory bowel disease of humans. The latter could suggest the zoonotic potential of MAP [15], although infection of humans with MAP and its association with Crohn's disease remains a controversial [16].

Paratuberculosis occurs in most part of the world and its incidence is rising from time to time [1]. It has been reported from many European countries, Oceania, Asia and African countries [17, 18]. The disease has also reported from the American continents [19–21]. Many studies have been done around the globe to determine the prevalence of MAP in cattle as well as in other species [1]. A recent study critically reviewed published data representing the prevalence of MAP in Europe and estimated the overall prevalence to be 20% [22]. The true prevalence among cattle was measured in studies conducted in France, Germany, Italy and Turkey. It has been suggested that the prevalence of MAP is at least 3–5% in several countries [22]. In the USA the infection is spread throughout the country and the herd prevalence is strongly associated with herd size. In 1996, the herd level prevalence of bovine paratuberculosis was reported to be 21.6% and within herd prevalence to be 40% as forty percent of herds with more than 300 heads was found to be infected [23].

In contrast to these industrialized countries, the exact sanitary situation of most African countries with regards to paratuberculosis is unknown but the occurrence of the diseases is suspected in most countries and the disease has been confirmed in a few countries of Africa. There are very few studies carried out to date, which leaves a big information gap on a very important disease of livestock. Only case reports and limited prevalence studies covering small areas in a few countries are available [24]. For example, studies were conducted in central and western Uganda on the epidemiology of paratuberculosis and detected diversified strains of Map [25, 26]. However, no comprehensive nationwide study was conducted on the prevalence paratuberculosis in Uganda. In Sudan, the disease was first diagnosed in goats and later in cattle and Map was first isolated Sudan in 1986. A recent study showed seropositive animals to MAP antibodies in 66% of the dairy farms in Khartoum State [27]. In Kenya, an earlier report indicated the detection of paratuberculosis in cattle from various herds in parts of western Kenya. Later, antibodies to MAP were found in 4.5% of sera cattle from 200 herds around Kabeete in central Kenya as well as in goats and camels in the coastal region of Kenya [28].

Prior to this study, Temesgen and Gemehu in 1995 reported a case of paratuberculosis from Ethiopia based on the history of diarrhea cases lasted for two years and clinical examination [29] although this observation was not supplemented with confirmatory diagnostic methods. Thus, even though there were reports of clinical cases of paratuberculosis from different veterinary clinics in Ethiopia, there was only a single seroprevalence study that was published so far [30]. Hence, well-designed epidemiological studies are required to be conducted on paratuberculosis in cattle in Ethiopia. Therefore, the present study was conducted to estimate the prevalence of paratuberculosis using gross and microscopic lesions in cattle slaughtered at ELFORA Abattoir, central Ethiopia.

## Material and methods

### Study area

The study was conducted from September 2013 to July 2014 at Bishoftu ELFORA export abattoir in central Ethiopia. ELFORA export abattoir is located at Debre Zeit Town 47 km southeast of Addis Ababa. Currently the abattoir is one of the modern export abattoirs in Ethiopia and is exporting meat of small ruminants to Middle East countries (Saudi Arabia, Dubai and Yemen) and African countries (Djibouti, Congo Brazzaville, Cote-d’ivoire and Egypt), But the cattle slaughtered in the ELFORA export abattoir are used for local consumption. During the study on average 400 and 500 sheep and goats respectively, were slaughtered at this abattoir per day. On the other hand, on average 70–85 cattle were slaughtered per week based on local market needs.

### Study animals

Animals used for the study were cattle slaughtered at ELFORA export abattoir. The study cattle were purchased from different zones of the country particularly Borana, Arsi, Bale, Gondar, Jimma and southern Ethiopia. All study animals were local breed (*Bos indicus)* and only males with variable body condition scores and age groups.

### Study design, sampling method and sample size determination

A cross-sectional study was carried out from September 2013 to July using abattoir based survey on apparently healthy cattle slaughtered to determine the prevalence of paratuberculosis. The abattoir was visited twice a week. Animals were included in the study using systematic random sampling method where only the first animal was chosen randomly. The sample size was determined by the formula described by Thrusfield [31]. The expected prevalence was set as 50% as there was no study conducted before in the study area. Therefore, the calculated sample size with a desired precision of 5% and 95% confidence interval was 384 animals. However, in order to increase our chances of finding several lesions for further histopathological examination, the carcasses of 400 animals were inspected.

### Postmortem examination and tissue sampling

Post mortem examination was performed on the digestive system (small and large intestine) and on the associated intestinal lymph nodes as previously described by Hailat [11]. All changes observed grossly such as mucosal corrugation, thickening, and hyperemia of the intestines were recorded. Furthermore, abnormalities of the intestinal lymph nodes including increase in size, change in shape and color were also recorded. Tissue samples were collected from grossly suspicious lesion of paratuberculosis. The lesions were trimmed into a smaller size of (4 mm to 1 cm) thickness, and fixed in 10% buffered formalin for histopathological examination.

### Histopathological examinations

Histopathological examination was performed according to a protocol described by Bancroft [32]. Tissue samples were sectioned into thin layers (4-5 μm) and stained with hematoxylin and eosin (H&E), as described by Prophet [33]. The H&E stained samples were observed under a compound microscope using 40X, 100X and 400X magnifications Histopathological lesions were recorded and graded as I, II, III and IV on the basis of the type and amount of cellular infiltration as described by other authors [11, 34]. Briefly, absence or presence of very few macrophages and lymphocytes without apparent thickening of the intestinal mucosa was recorded as negative. Lesions with lymphocytes with some macrophages with occasional or no epithelioid cells were considered as Grade I, while lesions with many macrophages with an increased number of lymphocytes, few scattered epithelioid cells and moderate Peyer’s patches proliferation and replacement of crypts was graded as II. Furthermore, a lesion with a prominent number of epithelioid cells in nests or scattered form with severe Peyer’s patches proliferation as well as with replaced crypts were graded as III. Finally, granulomatous lesions with multinucleated giant cells with or without epithelioid cells were considered as Grade IV.

### Data analysis

Data from gross examination and laboratory results were entered into Microsoft Excel 2010 spread sheets and the prevalence of bovine paratuberculosis was calculated in percentage. Animal prevalence was defined as the number of cattle found positive for paratuberculosis lesion per 100 animals examined.

## Results

### Prevalence of paratuberculosis on the basis of gross pathology

**Fig. 1:**
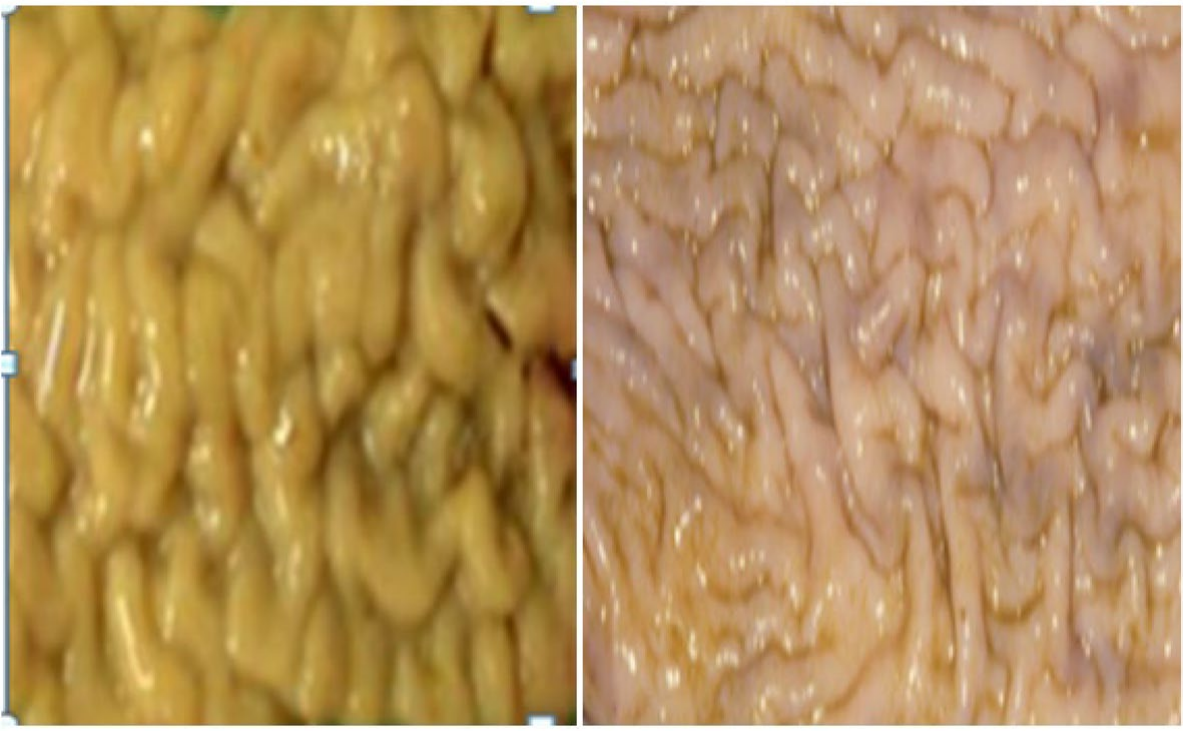
Gross lesion suggestive of paratuberculosis in the small intestine of apparently healthy cattle. The lesion was characterized by corrugation of the intestinal mucosa and thickening of the intestinal walls

**Fig. 2:**
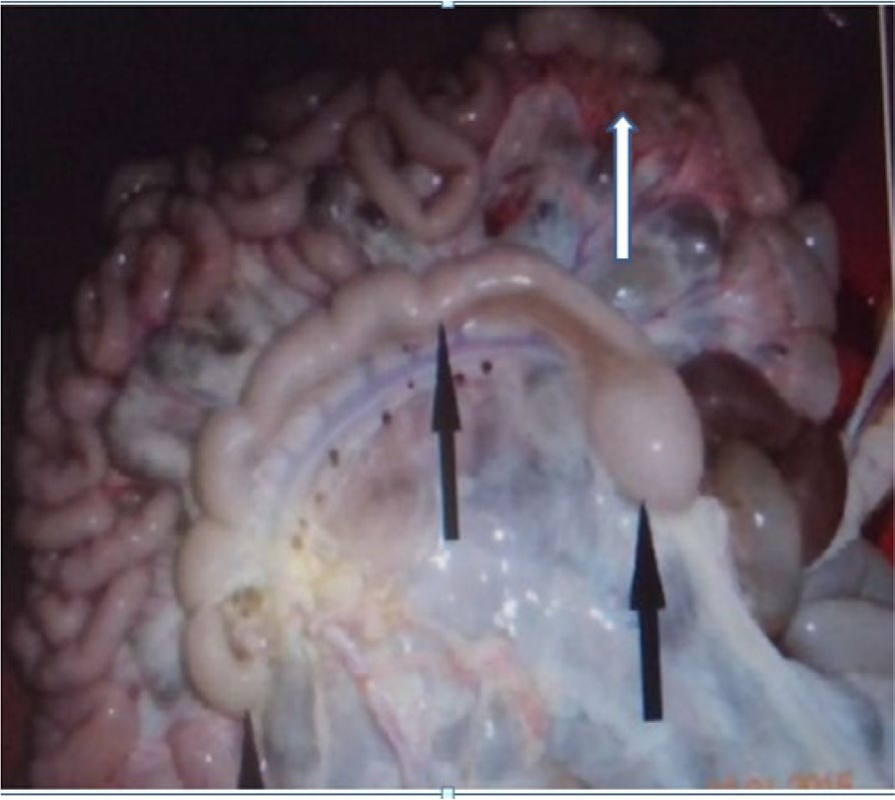
Gross lesions suggestive of paratuberculosis in the mesenteric lymph node of apparently healthy cattle. The lesion was characterized by enlarged, edematous (white arrow) and cording of the lymph nodes (black arrow)

The prevalence of paratuberculosis on the basis gross pathology was 11.25% (95% confidence, CI = 0.81–0.144). Thus, of the 400 cattle examined for the gross lesion of paratuberculosis, 45 cattle had intestines and/or lymph nodes with gross lesions compatible with the lesion of paratuberculosis. The gross lesions were characterized by thickening and corrugations of the intestinal mucosa, or enlargement of associated intestinal lymph nodes. Grossly, the intestinal wall was thickened in its different parts particularly at the ileoceacal junction. When thickened parts of the intestine were opened they showed corrugations and elevations which did not disappear on stretching (Fig. 1). The associated lymph nodes were severely enlarged, edematous and congested and they were connected to each other and appeared as cords (Fig. 2).

### Histopathological lesions

The prevalence of paratuberculosis on the basis of microscopic lesion was 2.0% (95% CI = 0.01, 0.039). Thus, the 45 cattle which were positive for gross lesion of paratuberculosis were further examined for microscopic lesion using Hematoxylin-Eoesin stained sectioned tissues of which eight animals had microscopic lesions consistent with the microscopic lesion of paratuberculosis. The microscopic lesions were characterized by an increase in the thickness and congestion of the intestinal mucosa due to inflammatory cells infiltrations. Out of the eight cattle with microscopic lesions, two cattle showed severe thickness and corrugation of the mucosa which were formed longitudinal and transverse ridges that could not be reduced by stretching (Fig. 1). Four animals showed mild thickening and characteristics transverse folding of the mucosa. Furthermore, the intestinal mucosa of two animals appeared diffusely opaque with fleshy thickening. The mesenteric lymph nodes of the ileum and ileoceacal valve area of all affected animals were severely enlarged, oedematous and congested (Fig. 2). On the serosal surface of the thickened intestine, prominent, white, enlarged and thickened tube-like lymphatic vessels were observed and the fat deposit on the mesentery was reduced or watery and showed serous atrophy of fat deposits.

**Fig. 3:**
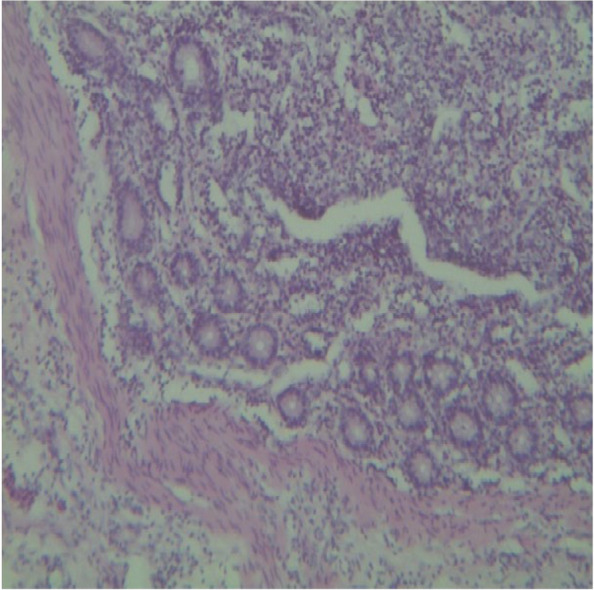
Microscopic lesions suggestive of paratuberculosis in the hematoxylin and eosin (H&E) stained section of the ileum of apparently healthy cattle. The lesion is characterized by diffuse Inflammatory cell infiltration in the mucosa and submucosa of the ileum. H&E stain 100X

**Fig. 4:**
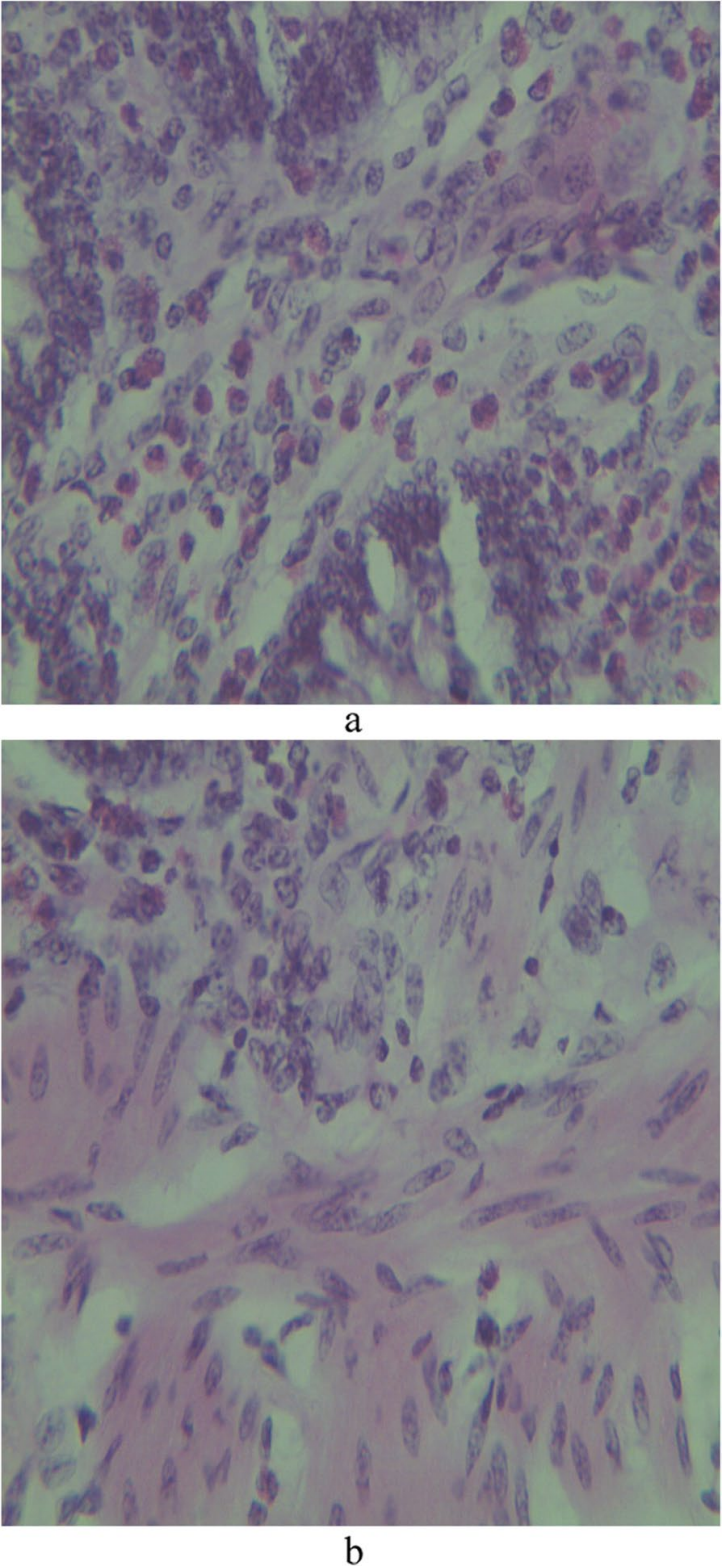
**a** Microscopic lesions suggestive of paratuberculosis in the hematoxylin and eosin (H&E) stained section of the ileum of apparently healthy cattle. The lesion is characterized by extensive numbers epithelioid cell infiltration. H&E stain 400X. **b** Microscopic lesions suggestive of paratuberculosis in the hematoxylin and eosin (H&E) stained section of the submucosa of apparently healthy cattle. The lesion is characterized by epithelioid cell infiltration forming nests or as a scattered form in the submucosa of the intestine. H&E stain 40X

**Fig. 5:**
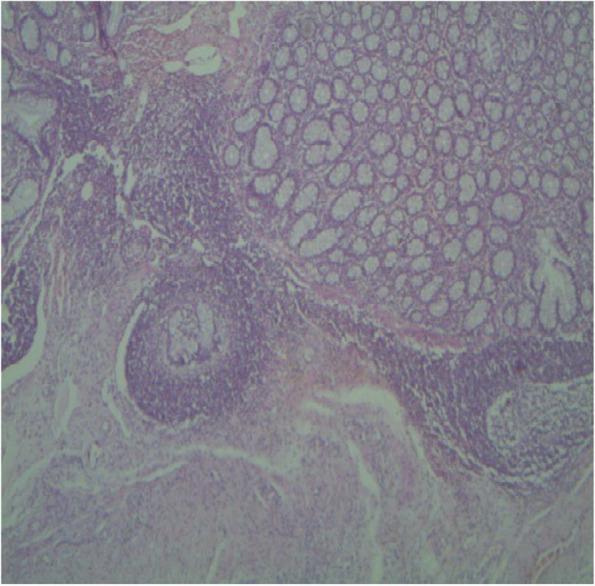
Microscopic lesions suggestive of paratuberculosis in the hematoxylin and eosin (H&E) stained section of the ileum of apparently healthy cattle. The lesion is characterized by Payer’s patches lymphoid hyperplasia and proliferation with extension towards the mucosa and crypt replacement. H&E stain 4X

**Fig. 6:**
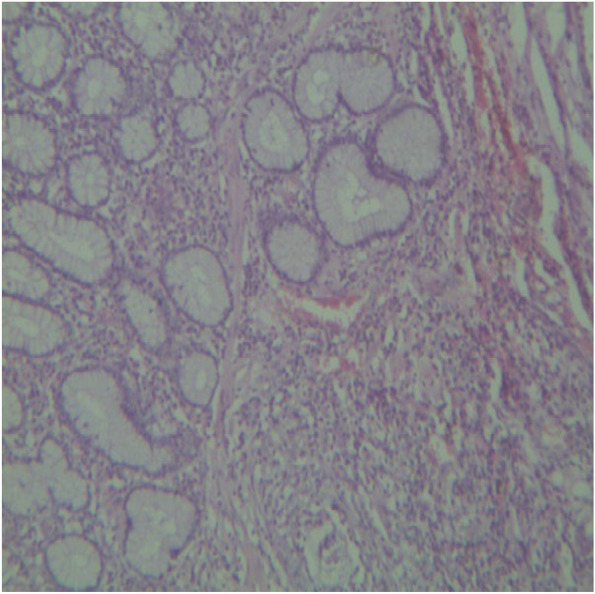
Microscopic lesions suggestive of paratuberculosis in the hematoxylin and eosin (H&E) stained section of the ileum of apparently healthy cattle. There is replacement of the crypts with macrophages and lymphocytes. H&E stain 10X

**Fig. 7:**
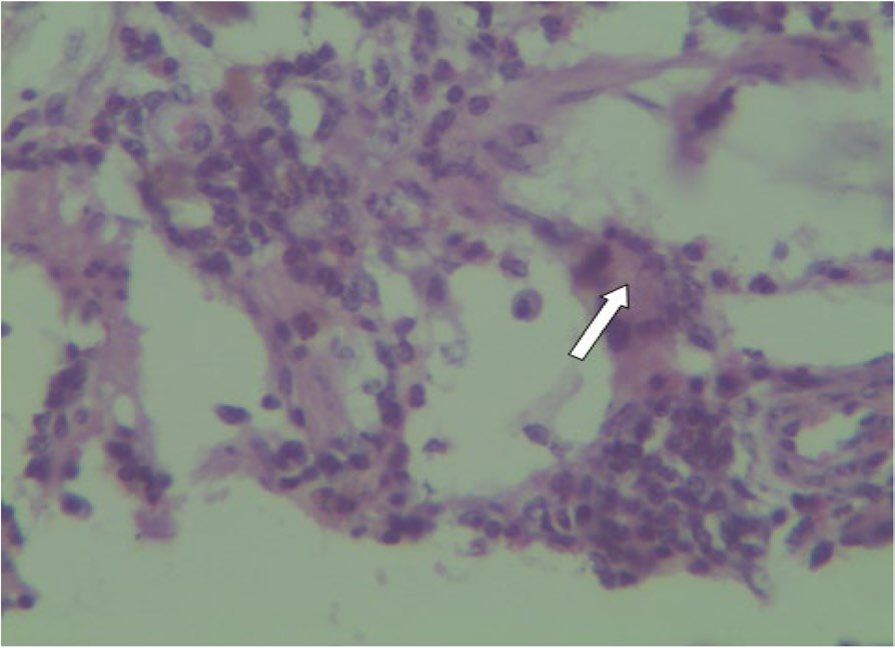
Microscopic lesions suggestive of paratuberculosis in the hematoxylin and eosin (H&E) stained section of the ileum of apparently healthy cattle. The lesion is characterized by presence of multinucleated giant cells in the sub mucosa of the intestinal section. H&E stain 40X

Microscopically, the mucosa and lamina propria of the ileum were diffusely infiltrated with mononuclear cells consisting primarily of lymphocytes with a few numbers of scattered macrophages (Fig. 3). In severe lesions, extensive numbers of inflammatory cells infiltrates mainly epithelioid cells with lymphocytes and plasma cells were seen (Fig. 4a). The epithelioid cells were present as a scattered or as nests (Fig. 4b). In Payer’s patches lymphoid hyperplasia and proliferation by macrophages and plasma cells with extension towards the mucosa were noted (Fig. 5). In most cases, the crypts were replaced by inflammatory cells especially macrophages and lymphocytes. Sometimes the Payer’s patch proliferation was replacing the crypts (Figs. 5 and 6). A small number of multinucleated giant cells were also seen in the affected tissues (Fig. 7). In some sections the villi were infiltrated with epithelioid cells and macrophages and they become short and thick as a result of this infiltration. Lesions observed in the mesenteric lymph nodes were consisted of diffuse infiltration of lymphocytes, macrophages and epithelioid cells with notable expansion towards paracortex. In cattle with sever lesions the epithelioid cells were found in nests throughout the lymph nodes.

Thus, out of the eight cattle with microscopic lesions suggestive of paratuberculosis 2 animals had grade I, 4 grade II and 2 grade III lesions. In grade III lesions typical granulomatous changes consisting of epithelioid macrophages and multinucleated giant cells were observed in the small intestines and their associated lymph nodes. Grade II lesions had the same pathological pattern of grade III, except that the severity of the lesions was moderate with few epithelioid cells, but there were more lymphocyte infiltrations. While the grade I lesions had less cellular infiltrations which consisted more of lymphocytes and some scattered macrophages.

## Discussion

In the present study, small intestines and associated lymph nodes of 400 apparently healthy cattle slaughtered at ELFORA export abattoir were examined for gross and microscopic lesions of paratuberculosis. The microscopic lesions were classified into four grades on the basis of type and number of cells infiltrated into the lesion (I-IV). The prevalence of paratuberculosis was estimated on the basis of gross as well as microscopic lesion of paratuberculosis.

The prevalence of paratuberculosis recorded in the present study on the basis gross lesion was 11.25%, which was similar with the prevalence of the study conducted earlier in Canada [35] while it was lower than the prevalence reported from Jordan and Uganda [11, 25]. On the other hand, it was higher than the prevalence reported from German and Mexico [36, 37]. The difference in the prevalence of paratuberculosis lesions could be due to variation in farm husbandry, age of the animals, implementation of control methods, immunological status of the host, feeding and farm hygiene [38].

Macroscopically a variety of gross lesions which were normally associated with paratuberculosis were observed in the ileum. Thickening and corrugations of the ileum were obvious in the last portion of the ileum particularly at the ileoceacal junction. In severely affected cases, there were diffuse thickenings along with transverse and longitudinal corrugations of the intestinal wall making irregular folds [39–42]. Congested, edematous, enlarged and corded mesenteric lymph nodes were observed on the ileum and ileoceacal junctions, which is in agreement with the reports made by other authors [11, 42].

The prevalence of paratuberculosis recorded in the present study was 2% on the basis of microscopic lesion. This microscopic lesion-based prevalence was comparable with the prevalence reported by the study conducted earlier in Pakistan [42], while it was higher than those reported by the other authors from Germen, Jordan, Mexico and Australia [11, 36, 37]. Microscopically, the thickness and corrugations occurred as a result of cellular infiltration in the mucosa and lamina propria as reported earlier [39]. The histopathological findings such as infiltration of the mucosa and lamina propria with lymphocytes, macrophages, replacement of the crypt with inflammatory cells, Peyer’s patches proliferation and extending towards the mucosa and the presence of multinucleated giant cells in the present study were consistent with those observed in small ruminants and cattle by other authors [11, 25, 34, 41–44]. Furthermore, the observation enlarged, edematous and congested mesenteric lymph nodes was similar with the observations made earlier by other authors [11, 25, 34].

The grade III lesion found in 2 cattle had diffuse infiltration of epithelioid cells, macrophages and multinucleated giant cells in the last portion of the small intestinal ileum and their associated lymph nodes. The grade III lesions observed in the present study corresponds most closely to those of the multibacillary form of paratuberculosis which is in agreement with the earlier by other authors [11, 44–48]. This form of paratuberculosis is thought to be the result of weak cellular immune response and strong humeral responses, which is common during the last stage of the disease [49].

Grade II lesions had the same pathological pattern of grade III, except that the severity of the lesions was moderate with few epithelioid cells, but there were more lymphocyte infiltrations. While the grade I lesions had less cellular infiltrations which was consisted more of lymphocytes and some scattered macrophages. This observation is consistent with the observation made earlier by other researcher [47]. Previous studies on experimental and natural cases of paratuberculosis in sheep and goat have indicated preponderance of lymphoid cells to epithelioid cells in the early paucibacillary cases, in which bacteria could not generally demonstrated [47, 50, 51]. Diffuse lymphocytic infiltrations has been reported in the early stages of other mycobacterial infections and correlated with strong cell-mediated immune responses mediated by different subpopulation of lymphocytes such as helper, cytotoxic and suppressor cell [44, 52, 53].

The study is not without limitation as it was based on gross and microscopic lesions of paratuberculosis confirmation of the diagnosis using identification of the etiologic agent (MAP) or it is nucleic acid using molecular typing was necessary although this could not be done because of resources.

## Conclusion

This study indicated the occurrence of paratuberculosis in cattle of Ethiopia on the basis of gross and microscopic lesions. This study was the first in pathologically detecting lesions consistent with the lesion of paratuberculosis although only one study reported the seroprevalence of paratuberculosis earlier. Thus, the result of the present study highlights the need for additional studies to establish its epidemiology and economic significance of partatuberculosis in the country.

## Data Availability

All the data supporting the results are included in the article.

## Abbreviations

MAP: *Mycobacterium avium* Subsp*. Paratuberculosis*
JD: Johne’s disease
MLN: Mesenteric lymph node
H&E: Hematoxylin and Eosin
